# Artificial Intelligence in Scoliosis: Current Applications and Future Directions

**DOI:** 10.3390/jcm12237382

**Published:** 2023-11-29

**Authors:** Haozhi Zhang, Changfa Huang, Daoyun Wang, Kuan Li, Xiao Han, Xin Chen, Zheng Li

**Affiliations:** 1 Department of Orthopaedics, Peking Union Medical College Hospital, Chinese Academy of Medical Sciences and Peking Union Medical College, Beijing 100730, China; pumc_zhanghaozhi@student.pumc.edu.cn (H.Z.);; 2 Department of Orthopedics, Beijing Chaoyang Hospital, Capital Medical University, Beijing 100020, China; 3 Department of Orthopedic Surgery, Beijing Jishuitan Hospital, Fourth Clinical College of Peking University, Jishuitan Orthopaedic College of Tsinghua University, Beijing 100035, China

**Keywords:** scoliosis, artificial intelligence, modern medicine, clinical practice

## Abstract

Scoliosis is a three-dimensional deformity of lateral bending and rotation of the spine. Artificial intelligence (AI) is a set of theories and techniques for studying artificial intelligence, which realizes machine intelligence by simulating and expanding human intelligence. With the continuous development of the multidisciplinary integration trend of modern medicine, artificial intelligence is used more and more in the diagnosis and treatment of scoliosis. Artificial intelligence has been widely used in the study of scoliosis and has penetrated into all fields of clinical practice of scoliosis. At present, artificial intelligence has shown good application prospects in early screening, diagnosis, treatment decision making, intraoperative operation, and prognosis prediction of scoliosis. This paper mainly summarizes the application of artificial intelligence in the clinical practice of scoliosis, and briefly introduces the AI model and its specific applications. In addition, this paper also discusses the limitations and future development of artificial intelligence. In the future, artificial intelligence will bring greater convenience to the diagnosis and treatment of scoliosis and provide better therapeutic effects for patients.

## 1. Introduction

Scoliosis refers to a three-dimensional deformity of the spine with lateral curvature and vertebral rotation, which covers some characteristics including rib hump, shoulder imbalance, waist asymmetry [1,2]. The International Scoliosis Research Society (ISRS) defines scoliosis as a deformity in which the Cobb measurement of the spine on the coronal plane exceeds 10° [3]. Scoliosis can be divided into congenital scoliosis, neuromuscular scoliosis, syndromic scoliosis, and idiopathic scoliosis, and it involves people of all ages such as children, adolescents, and adults [4]. In addition, spine surgeons have proposed numerous classification methods and standards based on the overall strategy, surgical methods, and fusion levels for treating scoliosis, including King classification [5], Lenke classification [6], and PUMC classification [7]. These classifications are widely recognized for their comprehensiveness and the valuable guidance they provide for determining treatment approaches. At present, the etiology and pathogenesis of idiopathic scoliosis, the most common type of scoliosis, remain unclear [8]. This lack of understanding contributes to the disease’s often undetected onset, leading to serious spinal deformities by the time it is diagnosed. Such deformities gravely affect the physical and mental health, as well as the quality of life, of patients. Untreated idiopathic scoliosis can worsen, resulting in increased curve progression, back pain, respiratory and cardiovascular issues, and neurological complications [9]. In view of the characteristics and complexity of scoliosis, early screening and diagnosis, progress prediction, and rehabilitation should be thoroughly considered [10].

**Figure 1:**
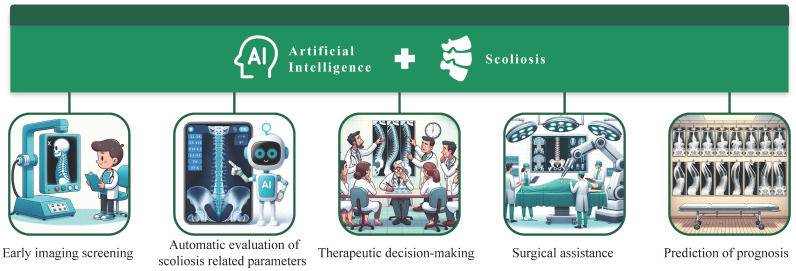
Application of artificial intelligence in various aspects of scoliosis diagnosis and treatment.

In recent years, artificial intelligence (AI) has developed rapidly and has been widely used in various medical fields. AI aims to imitate, extend, and expand human intelligence by studying artificial methods and technologies. This endeavor leads to the realization of machine intelligence [11]. The integration of AI with orthopedics, a field at the forefront of modern multidisciplinary cross-integration, showcases significant potential. Its clinical application value is gradually emerging after continuous development. Nowadays, the application and trend of AI in scoliosis mainly include the technology derived from machine learning (ML) and deep learning (DL), intelligent robots, and digital 3D printing technology [12]. As an advanced branch of AI, ML uses algorithms and machines to mine data features and learning rules from a large amount of data, without explicit programming [13]. In addition, the aim of DL is to simulate the neural network of the human brain and the ability for analysis and learning, which has become a new field in ML and neural network research [14]. At present, neural network technology based on ML and DL has shown some advantages in early imaging screening, auxiliary diagnosis, determination of the spinal parameters, determination of the diagnosis and treatment scheme, and prediction of the disease prognosis of scoliosis [15]. Moreover, orthopedic intelligent robots and AI-assisted 3D printing technologies also show unique advantages for improving the treatment efficiency of scoliosis [16]. Therefore, this article summarizes the application of AI in the diagnosis and treatment of scoliosis (Figure 1), in order to understand the current progress and future development trends of AI in scoliosis.

## 2. Early Imaging Screening

Early screening is the most effective way to find spinal deformity early, and early screening and diagnosis will contribute to the early intervention and treatment of spinal deformity [17]. Currently, imaging recognition has become an important field in the screening of spinal deformity. In view of the unique morphological and biomechanical characteristics of the spine, AI represented by ML has the ability to automatically obtain information in two-dimensional and three-dimensional images, which makes it possible to screen a wide range of spinal deformities.

In 2000, Jaremko et al. [18] firstly applied artificial neural network (ANN) to the study of scoliosis. The results showed that the average correct prediction rate of ANN was 60%, which was more accurate than the 34% of regression analysis, which indicates that this technology predicted rib deformity more accurately and allowed for evaluating the severity of scoliosis with the least use of harmful X-rays. Ramirez et al. [19] used a support vector machine (SVM) to process the human back topography image to evaluate the severity of idiopathic scoliosis. The results showed that the test accuracy of the SVM system reached 69–85%. Yang et al. [20] used the combined algorithm of Faster-RCNN and Res-Net to process unclothed back images to screen for AIS. The accuracy of this algorithm in detecting scoliosis and cases with curve ≥ 20° was better than that of human experts, and it could be potentially applied to routine scoliosis screening and as a periodic tool for monitoring disease progress without radiation exposure. However, initial radiographic examinations are necessary to rule out congenital scoliosis and syndromic scoliosis. Watanabe et al. [21] established a scoliosis screening system by evaluating spinal alignment, Cobb angle, and spinal rotation through Moiré topography. The system estimated the positions of 12 thoracic vertebrae, 5 lumbar vertebrae, and 17 spinous processes, as well as the vertebral rotation angle of each vertebra, through a convolutional neural network (CNN) with higher accuracy. In addition to processing back images using an algorithm of AI to screen scoliosis, Greer et al. [22] innovatively combined ultrasonic images and neural networks to achieve scoliosis screening for the population, and the system could also monitor the disease progress of scoliosis patients.

In the realm of AIS, the application of large language models (LLMs), such as advanced AI-based chatbots, is revolutionizing patient education and information dissemination. These AI-driven systems are adept at delivering personalized, comprehensible, and medically accurate information to patients and their families. The integration of LLMs in AIS management is particularly beneficial, given the condition’s complexity and the diverse treatment options available. LLMs provide a convenient and accessible platform for patients and families to address their questions, grasp the nuances of different treatment strategies, and participate actively in decision-making processes [23]. Furthermore, LLMs can tailor their responses to suit the user’s knowledge level and emotional state, ensuring a supportive and empathetic interaction [24]. This aspect is vital in AIS management, where patient engagement and comprehension are key factors in successful treatment outcomes. The incorporation of LLMs into AIS patient education programs is poised to augment patient autonomy, alleviate anxiety, and promote a participatory healthcare model, thereby leading to enhanced patient satisfaction and improved adherence to treatment plans.

Beyond educational purposes, LLMs also work well in assessing the severity of scoliosis. Contrastive language-image pretraining (CLIP) [25] is an AI model developed by OpenAI that combines text and image understanding in a joint embedding space. CLIP is designed to process and understand both text and images simultaneously, allowing it to perform tasks such as image classification, object detection, and image generation. Fabijan et al. [26] used 23 postural images of patients with severe scoliosis, evaluated by two neurosurgery experts. The images are fed into the CLIP system and the predictions obtained are compared with the actual data. The results show that the CLIP system can perform a basic evaluation of X-ray images showing severe scoliosis with a high sensitivity. Research suggests that in the future, open-source AI models specialized for image analysis, such as CLIP, may be commonly used to evaluate X-ray images of scoliosis.

The key to the early screening of scoliosis by AI is to obtain human trunk information. Currently, the methods of AI for the early screening of scoliosis are constantly being updated, and its reliability and accuracy are constantly improving. In addition to these advancements, it is crucial to implement regular evaluations and updates to these AI models to ensure their effectiveness in clinical settings. This involves rigorous performance monitoring to assess accuracy, sensitivity, and specificity in different populations and under varying clinical conditions. Moreover, model recalibration is essential to adjust algorithms in response to new findings or shifts in epidemiological trends. This recalibration should be an ongoing process, adapting the models to maintain their relevance and accuracy over time. Equally important is the incorporation of new data, which can come from recent clinical studies, patient demographics, or novel imaging techniques. By continually integrating this new information, AI models can evolve to become more robust and reflective of the current state of scoliosis presentations and treatments. It is believed that AI will become a common means for screening and monitoring scoliosis in the near future.

## 3. Automatic Evaluation of Scoliosis-Related Parameters

Quantitative analysis of scoliosis is a necessary step in the diagnosis and treatment of scoliosis [27]. The automatic analysis of related imaging indicators has the advantages of saving manpower and providing high accuracy, compared with manual measurement [28]. With the development of AI, more and more automatic analysis programs of spinal deformity images have been developed, presenting a more intelligent and accurate trend.

Automatic image analysis of spinal deformity mainly includes direct estimation and segmentation-based methods [29]. The acquisition of the Cobb angle in the direct estimation method is based on spinal X-ray images and the clinical measurement method. The spine feature points are identified on the X-ray, and then the subsequent parameters are calculated and the deflection angle is obtained, so as to calculate the Cobb angle. Weng et al. [30] used ResU-Net for the automatic measurement of the sagittal vertical axis of the spine. This model showed a high reliability for different types and degrees of deformity. BoostNet [31] was developed through the integration of a stand-alone deep CNN and a robust generative adversarial network, forming an ensemble network that effectively enhanced the denoising performance. On the basis of BoostNet, Wu et al. [32] proposed MVC-Net, which integrated the spine curvature of multi-view X-ray in anterior−posterior (AP) and lateral (LAT) positions, and could accurately and reliably evaluate Cobb angle and spinal landmarks. Wang et al. [33] further designed MVE-Net, which directly estimated Cobb angle from multi-view X-rays. Zhang et al. [34] proposed MPF-net, which effectively solved the problem of unclear identification of vertebral key points of LAT X-ray through the designed feature fusion module, which utilized the information in both AP and LAT X-rays for better performance, and further improved the accuracy of the automatic evaluation of the Cobb angle.

The segmentation-based method extracts the vertebral contour, and then measures scoliosis according to the segmentation results. Previous studies have developed a variety of segmentation-based methods for evaluating scoliosis, such as Hough transform [35,36], active contour models [37], customized filters [38], and an improved charged particle model [39]. Although these traditional segmentation methods have a low dependence on clinical measurement, they are still limited by some measurement shortcomings, such as the preference of user and vertebral selection. In recent years, deep learning methods have made remarkable progress in image recognition and segmentation, and several U-net models have been developed for spine segmentation and Cobb angle estimation [39,40,41]. Horng et al. [39] compared several U-Net models with the effect of vertebra X-ray image segmentation, and the results showed that Res U-Net had a better segmentation effect than U-Net and Dense U-Net. In addition, there was no significant difference in cobb angle measurement between these three U-Net models and clinicians. Recently, Zhao et al. [41] proposed a new U-Net model, which used the Inception block, Res block, and CBAM block to extract the multi-scale features of vertebrae, further improving the segmentation performance and the measurement accuracy of the Cobb angle.

In the realm of scoliosis diagnosis and treatment, AI has revolutionized the automatic evaluation of related parameters, particularly in quantitative analysis, which is crucial for accurate diagnosis and treatment planning. The advancement of AI has led to the development of various automatic analysis programs for spinal deformity images, showcasing a trend towards greater intelligence and precision. These include direct estimation methods like ResU-Net, BoostNet, MVC-Net, and MVE-Net, which have demonstrated a high reliability for measuring spinal parameters such as the Cobb angle from X-ray images. In parallel, segmentation-based methods, evolving from traditional techniques like Hough transform and active contour models to advanced deep learning approaches like various U-Net models, have significantly improved in vertebral segmentation and Cobb angle estimation. For instance, Horng et al. [39] and Zhao et al. [41] provided notable contributions with their U-Net models, enhancing segmentation accuracy and measurement precision. To maintain the effectiveness and relevance of these AI models, continuous performance monitoring is essential. This entails regularly assessing their accuracy and recalibrating algorithms based on new clinical data and imaging techniques. By incorporating such ongoing updates and evaluations, these AI models can sustain their accuracy and utility in the ever-evolving landscape of scoliosis diagnosis and treatment.

## 4. Therapeutic Decision-Making

The classification of scoliosis can help surgeons classify curve types and guide them to make treatment decisions [42]. Currently, the classification of scoliosis includes the King [5], Lenke [6], and PUMC [7] classifications. AI technology such as ML and DL can realize the rapid classification and diagnosis of scoliosis, and plays a positive role in making treatment decisions.

With the support of big data and image recognition technology, AI assists orthopedic surgeons to diagnose and treat diseases by learning massive knowledge and experience. Phan et al. [43] applied a decision tree to classify 72 patients with scoliosis, and the accuracy rate reached 92.9%. Chen et al. [44] designed a combined model of Faster R-CNN and ResNet to classify spinal images, which realized the accurate and rapid classification of scoliosis, thus contributing to the standardization and automation of surgical diagnosis.

Early prediction of the progression risk of scoliosis has significant clinical value, so as to help ensure that patients at risk of progression receive early supportive treatment and to strengthen treatment compliance. An AI algorithm can be applied to automatically predict the risk of scoliosis progression. Yahara et al. [45] developed a deep convolution neural network (DCNN) system to predict the curve progress of scoliosis by incorporating the source data of the X-rays of 58 AIS patients with three regions of interest (ROI): lung, abdomen, and whole spine. This model could predict the progress of scoliosis more accurately than spinal surgeons, with the highest accuracy of 69% and AUC of 0.7. Wang et al. [46] automatically predicted the progress of the AIS curve based on radiology and the CapsNet algorithm, which is helpful to guide the treatment strategy when visiting a doctor.

The procedure of surgical instrumentation for scoliosis is complicated, in which the choice of a fusion region is very challenging. Currently, the Lenke classification model is used in the surgical planning of spinal deformity to determine the appropriate fusion region. Mezghani et al. [47] trained self-organizing maps (SOMs) to determine the relationship between Lenke classification and fusion region selection from the database of 1776 AIS cases treated by surgery. The results showed that the overall consistency between them reached 88%, except for the near boundaries between Lenke maps. In addition, the SOM developed by the team bypassed the restrictions imposed by strict classification on the definition of curve types, thus guiding surgery [48].

Incorporating the prediction of surgical results into treatment decisions can improve patients’ satisfaction and reduce the occurrence and risk of reoperation. Pasha et al. [47] used the K-means algorithm to cluster and analyze the 3D spinal curve of AIS patients before, during, and 2 years after operation, which showed a good guiding role in treatment decision making and surgical operation. In addition, Pasha et al. [49] also cluster-analyzed the 3D spinal curve patterns of 371 AIS patients who underwent spinal fusion surgery before and 2 years after operation. The results showed that the 3D preoperative cluster classification had a higher accuracy than the Lenke classification in postoperative spinal alignment, and this classification system had potential application value in the prediction model of AIS surgical results [50]. Koller et al. [51] established a prediction model of postoperative spontaneous lumbar Cobb correction (SLCC) by using a logistic regression model, which contributed to surgical decision making during selective thoracic spinal fusion. In addition to determining the fusion levels and predicting the postoperative outcome, some AI methods have been developed to predict the progression of scoliosis, which helps doctors choose appropriate treatment measures. Carlo et al. [52] predicted the development of neuromuscular scoliosis in children with cerebral palsy based on a logistic regression algorithm, which could provide the basis for the treatment of patients. Cano et al. [53] utilized a neural network to extract the 3D structure of a patient’s spine and employed Independent Components Analysis [54] to predict the spine’s shape and changes from the first visit, enhancing the accuracy of predicting the curve progression. This approach guided the treatment effectively to prevent the curvature from progressing.

AI plays a pivotal role in therapeutic decision making in scoliosis, enhancing the classification, progression prediction, and surgical planning processes. AI facilitates rapid and accurate scoliosis classification, as seen in the works of Phan et al. [43] and Chen et al. [44], who employed decision trees and combined models of Faster R-CNN and ResNet, respectively. These classifications aid in making informed treatment decisions. Additionally, AI algorithms predict scoliosis progression, exemplified by Yahara et al.’s deep convolution neural network [45] and Wang et al.’s CapsNet algorithm [46], providing crucial early intervention insights. In surgical planning, AI models like self-organizing maps by Mezghani et al. [47] help determine appropriate fusion regions, offering a more flexible approach compared with traditional Lenke classification. Moreover, AI-assisted predictions of surgical outcomes, as researched by Pasha et al. [49], Koller et al. [51], Carlo et al. [52], and Cano et al. [52], contribute significantly to patient satisfaction and treatment strategies. To ensure these AI systems remain effective and relevant, continuous performance monitoring, model recalibration, and the integration of new clinical data are imperative. This ongoing process of evaluation and enhancement ensures AI models adapt to evolving medical knowledge and patient needs, solidifying their role in the dynamic landscape of scoliosis treatment.

## 5. Surgical Assistance

### 5.1. Insertion of Pedicle Screw

Pedicle screw insertion is a key step in scoliosis correction surgery. Pedicle localization and identification play an important role in screw insertion for spinal deformity. The AI algorithm contributes to the localization and identification of pedicles. Esfandiari et al. [55] established a DL framework based on fluoroscope for automatic segmentation and posed estimation of pedicle screws, which showed a good application effect, where the accuracy of pose estimation of this framework in clinical cases was 1.93 ± 0.64° and 1.92 ± 0.55 mm. Burström et al. [56] used an ML algorithm to construct an intraoperative 3D surgical navigation system, and the system could automatically identify the pedicle and provided suggestions for pedicle screw insertion. Further, the 3D prototype model and navigation system based on the AI algorithm can be used to guide surgeons to implant pedicle screws and improve its accuracy. Li et al. [57] utilized a rapid prototyping spinal model to insert screws using pedicle guide navigation. The intraoperative findings of this technique were completely consistent with the preoperative 3D reconstruction results, which improved the accuracy of pedicle screw insertion in complex scoliosis surgery. Zhang et al. [58] constructed a deformable 3D−2D registration framework based on preoperative CT and intraoperative long cross-sectional images. This method could automatically evaluate the overall spinal alignment and contribute to the accurate insertion of pedicle screws in the case of spinal deformation. Elmi-Terander et al. [59] compared the screw fixation accuracy of adding realistic surgical navigation (ARSN) and free hand (FH) technology, and ARSN showed a higher screw insertion accuracy, but both groups had no misplaced screws, and no differences in medial breaches were reported. Currently, the AI algorithm is also used to improve the product performance of pedicle screws, which can reduce the incidence of fixation failure in short-segment fixation. Amaritsakul et al. [60] carried out multi-objective optimization design of pedicle screws based on ANNs and a genetic algorithm (GA), and developed Pareto Optima, which balanced the bending strength and drawing strength. Finally, an ideal product with a high bending and drawability was developed using the AI algorithm. To summarize, AI algorithms could indeed be very useful for developing personalized implants based on patient-specific needs. By leveraging patient data and machine learning techniques, AI can predict and model optimal implant shapes, sizes, and materials that conform precisely to individual anatomical structures. This personalized approach could lead to implants that are more compatible with the patient’s body, potentially improving the success rates of surgeries, reducing recovery times, and minimizing the risk of postoperative complications.

### 5.2. Simulation of Deformity Correction and Intraoperative Monitoring

Correction simulation and intraoperative functional detection of spinal deformity can gain profit from the contribution of AI. By considering preoperative spinal alignment and possible complications during operation, it will help shorten the operation time and reduce the risk of blood loss, nerve injury, and infection. The bending of the rods during operation needs repeated comparison, which is not only time consuming, but also increases the risk of infection. Solla et al. [61] obviously improved the accuracy and time control of the operation through bending of the rods. Tachi et al. [62] developed a 4D surgical planning simulation system combined with pre-bent rods by studying the preoperative and postoperative information of 47 patients and 11 kinds of pre-bent rods. The predicted results of this system were significantly related to the actual postoperative spinal alignment after anatomical 4D spinal correction surgery, and the error between the simulated measured values and the actual values was within 5°. The AI algorithm can be used to predict the physiological changes of somatosensory evoked potentials. Fei et al. [63] combined the long-term and short-term memory of attention (LSTM) with CNNs, which could predict the somatosensory evoked potential (SEP) when physiological variables changed during scoliosis surgery, and realized the real-time monitoring of intraoperative neurological deficits.

### 5.3. Robotics in Scoliosis Surgery

In recent years, robot-assisted technology has been applied in scoliosis correction surgery. Orthopedic robots use intraoperative image guidance technology and surgical navigation data to map surgical space and plan surgical paths, which has the functions of active positioning and coordinated human−computer movement [64]. In spinal surgery, this technique uses a robotic arm to drill the trajectory of pedicle screws, which has the advantages of a high accuracy for screw implantation, low risk of radiation exposure, and less complications such as neurovascular injury during operation [65]. Chen et al. [66] compared the accuracy and safety of robot-assisted pedicle screw implantation and artificial pedicle screw implantation in AIS surgery, and found that the intraoperative blood loss, screw implantation time, and screw adjustment times of robot-assisted pedicle screw implantation were significantly less. Additionally, compared with traditional fluoroscopy-assisted technology and navigation technology, robot-assisted technology also showed better accuracy for pedicle screw placement in AIS surgery [67,68].

However, the robot device is controlled by the surgeon, and its surgical effect is influenced by the learning curve stage of the manipulator. Therefore, solid surgical skills and knowledge are still essential for surgeons to ensure the safety of patients.

## 6. Prediction of Prognosis

Employing machine learning algorithms to anticipate the prognosis and potential complications following scoliosis surgery can significantly enhance the assurance of patient recovery in the postoperative phase. Imaging complications of scoliosis patients mainly include proximal junctional kyphosis (PJK) and proximal junctional failure (PJF). Scheer et al. [69] used the C5.0 algorithm to build a decision tree set. This model could predict PJK and PJF in the perioperative period with an accuracy of 86.3% and an operating characteristic curve (AUC) of 0.89. Peng et al. [70] established a prognostic model for patients with Lenke 5 AIS based on the random forest trained by SMOTE. The accuracy of this model was 90.9% and AUC was 0.944, which was of great value for predicting the individual risk of PJK after AIS fusion surgery. Yagi et al. [71] predicted the risk of complications after Adult Spinal Deformity (ASD) surgery for 2 years based on decision tree analysis, and found that implant-related complications were the most common complications within 2 years after surgery. The accuracy of this model was 84%, and the AUC was 0.963. Pellisé et al. [72] employed a stochastic forest survival algorithm to forecast the likelihood of adverse events following surgery for ASD, revealing that the cumulative risk of significant complications in individual surgeries ranged from 3.9% to 74.1% over a two-year period. This research underscores the critical clinical relevance of utilizing AI algorithms for prognostic predictions. To further enhance this understanding, the study constructed two distinct models for each outcome, utilizing random survival forest algorithms. This approach emphasizes the crucial importance of developing precise and accurate prognostic models in clinical practice. Ames et al. [73] created a prediction model to simulate the possibility of achieving the minimum clinically significant difference in patient-reported outcomes (PROs) between 1 and 2 years after operation. The model found that the average improvement degree of patients with poor baseline PROs before operation was the highest, which had important clinical significance for postoperative care and rehabilitation. Additionally, the team developed a model based on the Scoliosis Research Society-22R (SRS-22R) questionnaire to forecast the scores 1 and 2 years post-surgery. This innovation is significant for enhancing our understanding of patients’ quality of life postoperatively. To our knowledge, this is the first study to focus on modeling the prediction of specific responses to the SRS-22R questionnaire at 1 and 2 years following deformity surgery. Predicting individual responses to this questionnaire can be particularly beneficial for personalized preoperative counseling, aligning with the contemporary trend of individualized medicine [74].

## 7. Future Directions of AI in Scoliosis

In recent years, AI has made remarkable progress in the diagnosis and treatment of scoliosis. In addition, AI has been gradually applied in case management and postoperative rehabilitation of patients. At present, AI technology is in the early stage of development, and there are still the following limitations and problems: ① AI has missed diagnosis and misdiagnosis in disease screening and diagnosis, and its accuracy is not 100% effective. ② Limited data and lack of multi-center verification in diverse clinical environments. ③ Lack of a perfect model integration platform, and lack of stability and limited generalization ability of the model. ④ Multi-professional collaboration to support and develop AI may pose economic challenges. ⑤ The execution rate of surgical robot programs is limited, and the operating systems are not fully intelligent. ⑥ AI involves patients’ data privacy and security, and its ethical issues cannot be ignored.

When evaluating the transformative potential of AI in the management of scoliosis, it is paramount to consider its impact on the standard of healthcare. AI methodologies, particularly advanced imaging algorithms and machine learning techniques, promise a paradigm shift in both diagnosis and treatment strategies. For instance, AI can enhance the accuracy of scoliosis detection and curve progression prediction, leading to more timely and personalized interventions. Moreover, AI’s ability to integrate and analyze vast datasets can aid in developing more effective treatment modalities, potentially reducing the need for invasive procedures such as surgery. However, the integration of AI into clinical practice also raises significant ethical considerations. The use of AI in medical decision making must ensure patient privacy, data security, and mitigate biases inherent in algorithmic processes. As highlighted in the article “*Ethical Issues of Artificial Intelligence in Medicine and Healthcare*” [75], there is a critical need for ethical frameworks that guide AI implementation in healthcare settings. These frameworks should address issues such as informed consent, transparency of AI-driven decisions, and equitable access to AI-enhanced medical care. By rigorously addressing these aspects, AI can be harnessed to not only advance the standard of care in scoliosis, but also to uphold the ethical integrity of medical practice.

In terms of models, the simulation system and future planning of AI need to be continuously optimized in many details, and clinical problems should be solved in combination with doctors’ clinical experience when designing the model. In terms of data, future AI algorithms need to be verified on the multi-center database platform to continuously improve the number and diversity of samples. Furthermore, it is imperative to underscore the importance of long-term studies for validating AI applications in diverse clinical environments. The effectiveness and safety of AI-driven interventions in scoliosis care must be demonstrated over extended periods and across varied healthcare settings. This is not only essential to ensure that these innovative technologies provide immediate clinical benefits, but also to maintain their efficacy and safety in the long term. Longitudinal studies will allow for the comprehensive assessment of AI’s impact on long-term patient outcomes, including quality of life and functional status post-treatment. Additionally, deploying AI tools in a variety of clinical settings, from specialized scoliosis centers to general hospitals, will enable the evaluation of their adaptability and generalizability across different patient populations and healthcare infrastructures. This approach aligns with the calls for rigorous validation in medical AI research, as is emphasized by Chen et al. [76]. By committing to extensive validation through long-term, multi-center studies, the medical community can ensure that AI tools used in scoliosis care are not only innovative, but that they are also reliable and universally beneficial.

The development of AI needs the joint efforts of bioinformaticians, computer scientists, data engineers, and doctors. Facing the rapid development of science and technology, doctors should know and be familiar with the theoretical knowledge and operating system of AI as much as possible, and gain the initiative in clinical use in the combination of AI and medical technology, so as to serve human society more effectively.

## 8. Conclusions

AI plays an important role in the clinical practice of scoliosis, which aims to assist the diagnosis of scoliosis and improve the treatment efficiency. At present, the application of AI in scoliosis mainly uses ML and DL algorithms for image segmentation to assist its diagnosis and treatment. In addition, the emergent spinal robotic technology also allows the surgical treatment of scoliosis to be more minimally invasive and intelligent, which can significantly improve its surgical outcomes. Although AI has made gratifying progress in early imaging screening, automatic evaluation of imaging parameters, treatment decision making, surgical assistance, and prognosis prediction of scoliosis, it still has some problems such as limited generalization ability and limited data sources. Looking ahead, several promising directions can further advance AI’s role in scoliosis management. First, there is a critical need for developing AI systems with enhanced generalization capabilities, capable of accurately functioning across diverse patient populations and varied clinical settings. This requires the collection and integration of large, varied datasets that reflect the spectrum of scoliosis presentations. Second, interdisciplinary collaborations between AI researchers, clinicians, and biomechanical engineers can foster innovative solutions, particularly in the realm of personalized medicine. Customized treatment plans based on AI-driven predictions could significantly improve patient outcomes. Third, exploring the potential of AI in patient monitoring and postoperative care can optimize long-term treatment strategies and enhance patient quality of life. Finally, as AI technology evolves, ethical considerations and the development of robust guidelines for its application in clinical practice must be a priority to ensure patient safety and data privacy. We believe that with the continuous development and optimization of AI, it can achieve revolutionary changes in the diagnosis and treatment of spinal surgery. In the future, AI will present more new methods and more reliable prediction models and infiltrate into all aspects of clinical practice related to scoliosis.

## Data Availability

No new data were created.
